# New R_2_-CHA_2_DS_2_-VASc score predicts no-reflow phenomenon and long-term prognosis in patients with ST-segment elevation myocardial infarction after primary percutaneous coronary intervention

**DOI:** 10.3389/fcvm.2022.899739

**Published:** 2022-10-13

**Authors:** Qinyao Zhang, Meirong Hu, Shumei Ma, Tiesheng Niu

**Affiliations:** Department of Cardiology, Shengjing Hospital of China Medical University, Shenyang, China

**Keywords:** R2-CHA2DS2-VASc score, ST-segment elevation myocardial infarction, primary percutaneous coronary intervention, no-reflow phenomenon, prognosis

## Abstract

**Aims:**

Evaluating the prognostic validity of new R_2_-CHA_2_DS_2_-VASc score for no-reflow phenomena and long-term prognosis in patients following primary percutaneous coronary intervention (PCI) with ST-elevation myocardial infarction (STEMI).

**Materials and methods:**

From January 2017 to December 2018, a total of 401 patients with STEMI were continuously enrolled. According to the cut-off value, the patients were separated into two groups: R_2_-CHA_2_DS_2_-VASc < 3 group (*n* = 275) and R_2_-CHA_2_DS_2_-VASc ≥ 3 group (*n* = 126).

**Results:**

With a sensitivity of 52.6% and a specificity of 73.1%, the optimal cut-off value for predicting no-reflow is R_2_-CHA_2_DS_2_-VASc ≥ 3. R_2_-CHA_2_DS_2_-VASc ≥ 3 as the ideal cut-off value for predicting major adverse cardiovascular events (MACE) with an area under the curve (AUC) of 0.781 [95% Confidence interval (CI): 0.738–0.801, *P* 0.001], a sensitivity of 50%, and a specificity of 91.1%. The incidence of MACE, death from all causes, and worsening heart failure was greater in the R_2_-CHA_2_DS_2_-VASc ≥ 3 group, although there was no significant difference in the incidence of repeated revascularisation procedures following PCI between the two groups. R_2_-CHA_2_DS_2_-VASc ≥ 3 was also an independent predictor of MACE (hazard ratio = 2.48, 95% confidence interval CI: 1.33–4.62, *P* = 0.04). Moreover, this score has a greater sensitivity (66.7%) and specificity (88.7%) for predicting the progression of heart failure.

**Conclusion:**

R_2_-CHA_2_DS_2_-VASc ≥ 3 was independently associated with no-reflow phenomenon and poor clinical outcomes for patients in STEMI after primary PCI.

## Introduction

Currently, primary percutaneous coronary intervention (PCI) remains the preferred treatment of choice for patients with STEMI. No-reflow phenomenon, a severe complication, occurred in approximately 5–15% of the patients following primary PCI (1, 2), and there were few effective treatment options. Moreover, the prognosis for STEMI patients remained dismal. Consequently, early risk stratification in STEMI patients to identify high-risk individuals was crucial. We anticipated discovering an indicator capable of predicting the no-reflow occurrence and stratifying the long-term prognosis of STEMI patients.

The CHA_2_DS_2_-VASc was a simple and extensively utilized score for determining the risk of stroke in atrial fibrillation patients (3). Numerous studies have demonstrated the association between the CHA2DS2-VASc score and the morbidity and prognosis of acute myocardial infarction patients (4–6). Renal function is a part of the new R2-CHA2DS2-VAsc score (7). We intend to determine if this score may more accurately predict the no-reflow phenomenon and long-term prognosis of STEMI patients following primary PCI.

## Materials and methods

This is a retrospective, single-center cohort study. From January 2017 to December 2018, 401 STEMI patients who underwent primary PCI at Shengjing Hospital of China Medical University were consecutively included. Inclusion criteria: patients were diagnosed with STEMI according to the 2017 diagnosis and treatment guideline for ST-segment elevation myocardial infarction (8). Exclusion criteria: severe liver dysfunction, cancer, hematological disorders, systemic immunological diseases and onset time > 24 h. Our ethics committee at Shengjing Hospital of China Medical University authorized this study(2019PS602K). Before participating in the study, all individuals gave their informed consent.

All hospitalized patients had standard blood tests. Before primary PCI, all patients received a loading dose of aspirin 300 mg, clopidogrel 300 mg, or ticagrelor 180 mg. During the procedure, the patients were administered 100 U/kg of heparin and Tirofiban, and all procedures were performed by senior physicians using conventional procedures. Our hospital information system had the patients’ baseline characteristics and angiographic features. Blinded to the study, three cardiologists assessed the thrombolysis in myocardial infarction (TIMI) flow grade.

CHA_2_DS_2_-VASc score including heart failure (C), hypertension (H), diabetes (D), age 65–74 years (A), peripheral vascular disease (V), and female (Sc) each earned 1 point, while Age ≥ 75 years old (A) and stroke or TIA (S) were recorded as 2 points. The R_2_-CHA_2_DS_2_-VASc score was derived by adding 2 extra points for renal function impairment, which was defined as estimated glomerular filtration rate (eGFR) ≤ 60 ml/min/1.73 m^2^ using the MDRD formula. TIMI flow grades are defined as follows: grade 0, the contrast agent cannot pass through the vascular occlusion; grade 1, a small amount of contrast agent can pass through the lesion but cannot fill the distal blood vessel; grade 2, the contrast agent can fill the distal blood vessel, but the filling rate is slower; and grade 3, the contrast agent can fill the distal blood vessels rapidly and completely. No-reflow phenomenon is defined as TIMI blood flow grade remaining ≤ grade 2 following revascularization of the culprit lesion in the absence of coronary spasm, vascular dissection, and thromboembolism (9).

All registered patients received telephone follow-up. Major adverse cardiovascular events (MACE) including cardiovascular death, repeated revascularisation procedures following PCI (stent thrombosis or recurrent myocardial infarction) and worsening heart failure (new-onset heart failure or previous heart failure acute episodes) were the primary endpoints. Secondary endpoint included all-cause death, repeated revascularisation procedures after PCI or worsening heart failure.

## Statistical analysis

SPSS 22.0 software was used for the analysis. Normal distribution measurement data were expressed as mean ± standard deviation, and the *t*-test was used to compare groups; non-normal distribution measurement data were expressed as median (interquartile range), and the Mann-Whitney *U*-test was utilized. The enumeration data were reported as numbers (percentages), and chi-square tests are used to compare groups. Using the receiver operating characteristic (ROC) curve was used to analyze the predictive power and the optimal cut-off value of the R_2_-CHA_2_DS_2_-VASc score for no-reflow phenomenon and long-term MACE were determined. The Kaplan-Meier survival curve method and the log-rank test were employed to compare survival rates between the two groups, respectively. The independent risk variables of no-reflow phenomenon after primary PCI were analyzed using logistic regression, and the influence of R2-CHA2DS2-VASc score on patient prognosis was determined using the Cox proportional hazards model. *P*<0.05 was regarded as statistically significant using a two-sided test.

## Results

**FIGURE 1:**
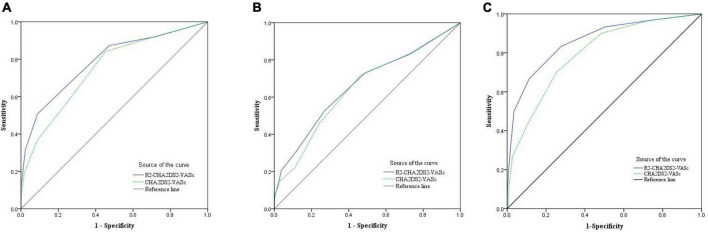
The ROC curve was used to demonstrate the distinction between the R2-CHA2DS2-VASc score and the CHA2DS2-VASc score and to calculate the cut-off value, sensitivity, and specificity of the R2-CHA2DS2-VASc score in **(A)** predicting long-term MACE. **(B)** Predicting no-reflow phenomenon. **(C)** Predicting worsen heart failure.

In Figure 1A, the ROC curve demonstrated the significance of the R_2_-CHA_2_DS_2_-VASc score in predicting long-term prognosis of STEMI patients following primary PCI. The area under the curve (AUC) of the R_2_-CHA_2_DS_2_-VASc score to predict MACE was 0.781 (95% CI: 0.738–0.821, *P* < 0.001), and with a sensitivity of 50% and a specificity of 91.1%. According the cut-off value, we classified the 401 patients into two groups: R_2_-CHA_2_DS_2_-VASc < 3 group and R_2_-CHA_2_DS_2_-VASc ≥ 3 group.

Table 1 summarized the baseline characteristics of the patients. Patients in R_2_-CHA_2_DS_2_-VASc ≥ 3 group tended to be older, predominantly male, and more likely to have a history of diabetes, hypertension, vascular disease, and stroke. However, there was no significant difference in the history of heart failure between the two groups. Initial creatine kinase-MB, total cholesterol, high-density lipoprotein cholesterol, low-density lipoprotein cholesterol, cardiac troponin I, leukocytes, platelets and creatinine values did not differ across groups. Compared to the R2-CHA2DS2-VASc<3 group, the R2-CHA2DS2-VASc ≥ 3 group had lower eGFR and hemoglobin levels, as well as higher Initial NT-pro brain natriuretic peptide and glycosylated hemoglobin A1c levels. The R_2_-CHA_2_DS_2_-VASc ≥ 3 group exhibited a higher Killip class, greater multivessel disease on angiography, and a large incidence of no-reflow phenomenon.

**TABLE 1:** Baseline clinical characteristics.

Variables	R_2_-CHA_2_DS_2_-VASc < 3 (*n* = 275)	R_2_-CHA_2_DS_2_-VASc ≥ 3 (*n* = 126)	*P*-value
Age, years	56.2 ± 10.8	67.4 ± 10.1	**< 0.001**
Male	240 (87.3%)	59 (46.8%)	**< 0.001**
Hypertension	83 (30.2%)	89 (70.6%)	**< 0.001**
Diabetes mellitus	44 (16.0%)	51 (40.5%)	**< 0.001**
History of heart failure	3 (1.1%)	1 (0.8%)	0.781
History of stroke/TIA	5 (1.8%)	40 (31.7%)	**< 0.001**
Vascular disease	25 (9.1%)	35 (27.8%)	**< 0.001**
Smoking	186 (67.6%)	50 (39.7%)	**< 0.001**
Leukocyte, × 10^9^/L	8.2 ± 3.5	7.9 ± 3.2	0.409
Hemoglobin, g/L	144.75 ± 14.2	132.08 ± 15.99	**< 0.001**
Platelets, × 10^9^/L	217.0 ± 56.5	218.41 ± 58.7	0.822
eGFR, ml/min/1.73 m^2^	100.76 ± 19.4	85.79 ± 28.46	**< 0.001**
SBP, mmHg	120.6 ± 18.2	120.6 ± 20.4	0.972
DBP, mmHg	76.7 ± 12.2	74.4 ± 13.0	0.094
HR, beats/min	77.5 ± 13.1	77.72 ± 14.9	0.889
TC, mmol/L	4.7 ± 1.1	4.6 ± 1.1	0.92
HDL-C, mmol/L	1.03 ± 0.28	1.02 ± 0.27	0.749
LDL-C, mmol/L	2.97 ± 0.93	2.95 ± 0.88	0.807
Creatinine, μmol/L	69.8 ± 16.0	74.6 ± 27.9	0.072
HbA1c,%	6.2 ± 1.3	6.6 ± 1.6	**0.003**
Initial cTnI, ng/L	0.14 (0.02,0.86)	0.27 (0.02,1.40)	0.281
Initial CK-MB, ng/L	15.5 (3.5,45.5)	3.4 (17.0,73.3)	0.450
Initial NT-pro BNP, pg/mL	66.95 (134.9,369)	121 (343,944)	**< 0.001**
CHA_2_DS_2_-VASc score	1.0 ± 0.8	3.5 ± 1.1	**< 0.001**
R_2_-CHA_2_DS_2_-VASc score	1.0 ± 0.9	3.9 ± 1.2	**< 0.001**
Killip class on admission			**< 0.001**
1	232 (84.4%)	86 (68.3%)	
>1	43 (15.6%)	40 (31.7%)	
Multivessel disease	124 (45.1%)	76 (60.3%)	**0.005**
TIMI flow grade before PCI			0.104
≤2	268 (97.5%)	126 (100%)	
>3	7 (2.5%)	0 (0%)	
Culprit artery			0.266
LM	0 (0)	0 (0)	
LAD	134 ± 48.7	71 ± 56.3	
LCX	63 ± 22.9	21 ± 16.7	
RCA	78 ± 28.4	34 ± 27.0	
Stent diameter	3.3 ± 0.4	3.2 ± 0.4	
Number of stents	1.5 ± 0.6	1.4 ± 0.6	0.468
No-reflow phenomenon	37 (13.5%)	41 (32.5%)	**< 0.001**

Data are presented as n (%), median (IQR), or mean ± SD. eGFR, estimated glomerular filtration rate; TIA, transient ischemic attack; SBP, systolic blood pressure; DBP, diastolic blood pressure; HR, heart rate; HbA1c, glycosylated hemoglobin A1c; TC, total cholesterol; HDL-C, high-density lipoprotein cholesterol; LDL-C, low density lipoprotein cholesterol; cTnI, cardiac troponin I; CK-MB, creatine kinase-myocardial band; NT-pro BNP, NT-pro brain natriuretic peptide; TIMI, thrombolysis in myocardial infarction; LAD, left anterior descending; LCX, left circumflex; RCA, right coronary artery; LM, left main.

Bold values means *P* < 0.05.

Analysis using univariate logistic revealed that R_2_-CHA_2_DS_2_-VASc ≥ 3, initial NT-pro BNP and killip class > 1 can predict the absence of reflow. R2-CHA2DS2-VASc 3 (OR = 2.75, 95% CI: 1.64–4.71, *P* 0.001) and Killip class > 1 (OR = 2.08, 95% CI: 1.12–3.08, P = 0.012) remained independent predictors in Table 2 based on the results of multivariate logistic regression.

**TABLE 2:** Results of the univariate and multivariate regression analyses for the predictors of the no-reflow phenomenon.

Variables	Unadjusted OR (95% CI)	*P*-value	Adjusted OR (95% CI)	*P*-value
R_2_-CHA_2_DS_2_-VASc ≥ 3	3.10 (1.87–5.16)	**< 0.001**	2.75 (1.61–4.71)	**< 0.001**
Initial NT-pro BNP	1.00 (1.00–1.00)	**0.015**	1.00 (1.00–1.00)	0.504
Killip class > 1	2.52 (1.46–4.37)	**0.001**	2.08 (1.12–3.87)	**0.021**
Multivessel disease	0.68 (0.41–1.12)	0.125	-	−
HbA1c	1.15 (0.98–1.35)	0.094	-	−
Hemoglobin	0.99 (0.98–1.01)	0.378	-	−
Stent diameters	0.93 (0.53–1.62)	0.792		

NT-proBNP, NT-pro brain natriuretic peptide; HbA1c, glycosylated hemoglobin A1c; OR, odds ratio; CI, confidence interval.

Bold values means *P* < 0.05.

Table 3 displays the adverse clinical outcomes that occurred after a median follow-up time of 22 (Q1-Q3: 16–28) months. Patients in the R2-CHA2DS2-VASc3 group experienced a total of 24 episodes of MACE, including 2 cases of cardiovascular death, 7 cases of worsening heart failure, and 15 cases of repeated revascularisation procedures following PCI. In R_2_-CHA_2_DS_2_-VASc ≥ 3 group, there were 40 cases of cardiovascular death, 23 cases of MACE, including eight cases of cardiovascular death, 23 cases of worsening heart failure, and nine cases of repeated revascularisation procedures following PCI.

**TABLE 3:** Long-term follow-up outcomes.

Variables	R_2_-CHA_2_DS_2_-VASc < 3 (*n* = 275)	R_2_-CHA_2_DS_2_-VASc ≥ 3 (*n* = 126)	*P*-value
All-cause mortality	6 (2.2%)	11 (8.7%)	**0.005**
Cardiovascular mortality	2 (0.72%)	8 (6.3%)	**< 0.001**
Worsening Heart failure	7 (2.5%)	23 (18.3%)	**< 0.001**
Repeated revascularisation procedures after PCI	15 (4.7%)	9 (7.1%)	0.348
MACE	24 (8.7%)	40 (31.7%)	**< 0.001**

MACE, major adverse cardiac events; PCI, percutaneous coronary intervention.

Bold values means *P* < 0.05.

In addition, we discovered that the R_2_-CHA_2_DS_2_-VASc score predicted MACE better than the CHA_2_DS_2_-VASc score (*Z* = 2.88, 95% CI: 0.013–0.069, *P* = 0.004) (Figure 1A). Figure 1B demonstrated the predictive usefulness of the R_2_-CHA_2_DS_2_-VASc score for the phenomenon of on-reflow. The optimal cut-off value is R_2_-CHA_2_DS_2_-VASc ≥ 3 with a sensitivity of 52.6% and a specificity of 73.1%. According to the Delong test, there was no statistically significant differences between the R_2_-CHA_2_DS_2_-VASc and CHA_2_DS_2_-VASc scores in their capacity to predict no-reflow phenomenon (*P* = 0.159), with a sensitivity of 66.7% and specificity of 88.8% (Figure 1C). In terms of predicting worsening heart failure, the R2-CHA2DS2-VASc score was also superior to the CHA2DS2-VASc score (*Z* = 2.92, 95% CI: 0.021–0.108, *P* = 0.0035).

**FIGURE 2:**
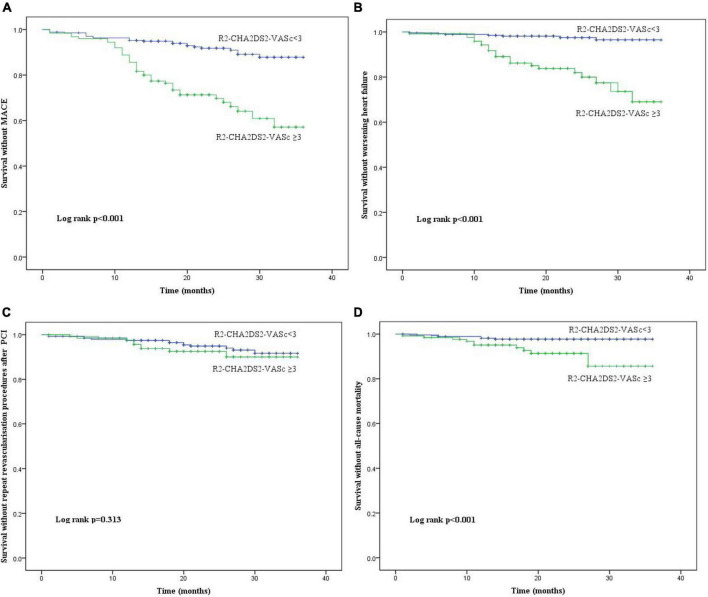
The Kaplan-Meier curves of the cumulative incidence of **(A)** MACE, **(B)** worsening heart failure, **(C)** repeated revascularisation procedures after PCI and **(D)** all R_2_-CHA_2_DS_2_-VASc score groups. MACE, major adverse cardiac events; PCI, percutaneous coronary intervention.

In terms of MACE, worsening heart failure, and all-cause mortality, there were statistically significant differences between the two groups; however, was no difference in repeat revascularisation procedures after PCI. The Kaplan-Meier method revealed that the risk of MACE, worsening heart failure, and all-cause mortality was significantly higher in R_2_-CHA_2_DS_2_-VASc ≥ 3 group than in the R_2_-CHA_2_DS_2_-VASc < 3 group (*P* < 0.001, Figures 2A,B,D), whereas the risk of repeated revascularisation procedures after PCI was not statistically different between the two groups (*P* = 0.313, Figure 2C). Table 4 demonstrates that R2-CHA2DS2-VASc 3, Killip class > 1, hemoglobin, and no-reflow phenomenon are independent risk factors for MACE (*P* < 0.05).

**TABLE 4:** Univariate and multivariate cox regression of long-term MACE.

Variables	Univariate HR value (95% CI)	*P*-value	Multivariate HR value (95% CI)	*P*-value
R_2_-CHA_2_DS_2_-VASc ≥ 3	4.19 (2.52–6.96)	**< 0.001**	2.34 (1.31–4.18)	**0.004**
Initial NT-pro BNP	1.00 (1.00–1.00)	**< 0.001**	1.00 (1.00–1.00)	0.316
Killip class > 1	2.99 (1.81–4.96)	**< 0.001**	1.84 (1.04–3.25)	**0.037**
Hemoglobin	0.97 (0.95–0.98)	**< 0.001**	0.98 (0.96–0.99)	**0.01**
No-reflow phenomenon	3.25 (1.96–5.40)	**< 0.001**	2.21 (1.27–3.84)	**0.005**
HbA1c	0.95 (0.79–1.14)	0.56	-	−

NT-proBNP, NT-pro brain natriuretic peptide; HbA1c, glycosylated hemoglobin A1c.

Bold values means *P* < 0.05.

## Discussion

The study’s key findings were as follows: (1) the R_2_-CHA_2_DS_2_-VASc score had relatively poor predictive value for no-reflow phenomenon, nevertheless, patients with R_2_-CHA_2_DS_2_-VASc ≥ 3 had a remarkable increase in long-term bad clinical outcome, particularly increasing heart failure, and (2) R_2_-CHA_2_DS_2_-VASc ≥ 3 was an independent predictor for long-term prognosis and no-reflow phenomenon in patients with STEMI.

The CHA_2_DS_2_-VASc score can be used to predict adverse cardiovascular events, death and other clinical outcomes, with the exception of stroke prediction in patients with atrial fibrillation (10–13). eGFR can be used for risk stratification in coronary artery disease patients (14, 15). Therefore, we believe that the addition of eGFR to the CHA2DS2-VASc score will improve the ability to predict complications and prognosis in STEMI patients.

No-reflow phenomenon is one of the most significant problems following primary PCI. No-reflow has been demonstrated to exacerbate myocardial ischemia, enlarge the region of myocardial infarction, and increase the incidence of heart failure, which is a predictor of both short- and long-term bad prognosis (16–18). The pathophysiological mechanism of the no-reflow is not entirely understood, but it is may be related to ischemia-reperfusion injury and distal vascular embolism (19). Among 428 consecutive patients with non-ST-segment elevation myocardial infarction, the CHA_2_DS_2_-VASc score in the no-reflow group was substantially higher than that in the normal flow group. The high CHA_2_DS_2_-VASc score was an independent predictor of no-reflow and the optimal cutoff value is 3 with 80.9% sensitivity and 74.6% specificity (20). Our investigation indicated that the R_2_-CHA_2_DS_2_-VASc score was independent predictor of no-reflow, with a sensitivity of 52.6% and a specificity of 73.1% for R2-CHA2DS2-VASc ≥ 3. In this study, the R_2_-CHA_2_DS_2_-VASc score had weak ability to predict no-reflow. This may be owing to the inclusion of all patients with STEMI in this study, or it may be due to the fact that the occurrence of no reflow is more related to the severe thrombotic burden, surgical procedures, etc.

We were not surprised that the CHA_2_DS_2_-VASc score can independently predict the occurrence of long-term adverse clinical outcome in STEMI patients, as numerous prior studies have demonstrated that each component of the CHA_2_DS_2_-VASc score influences the prognosis of patients with acute coronary syndrome (21, 22). Peng et al. found that MACE during hospitalization and long-term follow-up increased when the CHA_2_DS_2_-VASc score increased, and the CHA_2_DS_2_-VASc score exhibited independent predictive value for MACE (3). The other study demonstrated that the predictive value of the CHA2DS2-VASc score for all-cause mortality and cardiovascular mortality is marginally greater than that of the GRACE score (23). Our investigation demonstrated that R_2_-CHA_2_DS_2_-VASc ≥ 3 is the optimum cut-off value for predicting MACE with a sensitivity of 50% and a specificity of 91.1%. The Kaplan-Meier survival analysis of survival revealed that STEMI patients with R2-CHA2DS2-VASc 3 were more likely to experience MACE, all-cause death, and worsening heart failure; however, the incidence of repeated revascularisation procedures after PCI was not statistically different between the two groups (*P* = 0.313). This may be due to the correlation between in-stent restenosis and smoking, smaller stent diameter, longer lesions, and stent metal allergy.

This study demonstrated that a variety of risk factors, including hypertension, hyperglycemia, renal dysfunction, and heart failure, may contribute to an increase PCI complications and a worse prognosis over the long term. R2-CHA2DS2-VASc score is not a very good predictor of no-reflow phenomenon. However, according to the results of the study, it is also suggested that avoiding too high or too low blood pressure, avoiding stress hyperglycemia, and correcting acute heart failure may decrease the occurrence of no-reflow after PCI.

In addition, people at high risk for myocardial infarction can improve their prognosis by controlling their blood pressure and blood glucose levels. Angiotensin receptor enkephalinase inhibitor (ARNI) and sodium-dependent glucose transporters 2 inhibitor (SGLT2i) are two novel medications for heart failure that reduce blood pressure and blood sugar, respectively. Further investigation is required to determine whether ARNI and SGLT2i can reduce PCI complications and enhance prognosis in individuals with ST-elevation myocardial infarction (STEMI).

## Conclusion

Due to its excellent specificity, this score can be utilized as a risk stratification indication for poor prognosis, as determined by our research. I believe this score has a lot of utility, especially for forecasting deteriorating heart failure.

## Study limitations

This study also contains the following flaws: Firstly, this is a retrospective study with inherent biases in its design. Second, the number of patients included in this single-center trial is rather limited. As the data were primarily derived from a review of the prior clinical history of patients in an acute clinical environment, bias may also exist. The clinical value of the R_2_-CHA_2_DS_2_-VASc score must be proven by a prospective multicenter study with a large sample size.

## Data availability statement

The raw data supporting the conclusions of this article will be made available by the authors, without undue reservation.

## Ethics statement

The studies involving human participants were reviewed and approved by ethics committee of Shengjing Hospital of China Medical University. The patients/participants provided their written informed consent to participate in this study.

## Author contributions

QZ: concept, analysis, literature search, and writing. SM and TN: design and supervision. QZ and MH: materials and data. TN: critical revision. All authors contributed to the article and approved the submitted version.
